# Critical evaluation of the Illumina MethylationEPIC BeadChip microarray for whole-genome DNA methylation profiling

**DOI:** 10.1186/s13059-016-1066-1

**Published:** 2016-10-07

**Authors:** Ruth Pidsley, Elena Zotenko, Timothy J. Peters, Mitchell G. Lawrence, Gail P. Risbridger, Peter Molloy, Susan Van Djik, Beverly Muhlhausler, Clare Stirzaker, Susan J. Clark

**Affiliations:** 1 Epigenetics Research Laboratory, Genomics and Epigenetics Division, Garvan Institute of Medical Research, 384 Victoria St, Darlinghurst, Sydney, 2010 NSW Australia; 2 St Vincent’s Clinical School, University of NSW, Sydney, 2010 NSW Australia; 3 Prostate Research Group, Department of Anatomy and Developmental Biology, Biomedicine Discovery Institute, Monash Partners Comprehensive Cancer Consortium, Monash University, Clayton, Melbourne, VIC 3800 Australia; 4 CSIRO, Health and Biosecurity, PO Box 52, North Ryde, NSW 1670 Australia; 5 FOODplus Research Centre, Department of Food and Wine Science, School of Agriculture Food and Wine, Waite Campus, The University of Adelaide, Adelaide, SA Australia; 6 Child Nutrition Research Centre, South Australian Health and Medical Research Institute, Adelaide, SA Australia

**Keywords:** EPIC, DNA methylation, HM450, Whole-genome bisulphite sequencing (WGBS), Microarray, Enhancers, Validation

## Abstract

**Background:**

In recent years the Illumina HumanMethylation450 (HM450) BeadChip has provided a user-friendly platform to profile DNA methylation in human samples. However, HM450 lacked coverage of distal regulatory elements. Illumina have now released the MethylationEPIC (EPIC) BeadChip, with new content specifically designed to target these regions. We have used HM450 and whole-genome bisulphite sequencing (WGBS) to perform a critical evaluation of the new EPIC array platform.

**Results:**

EPIC covers over 850,000 CpG sites, including >90 % of the CpGs from the HM450 and an additional 413,743 CpGs. Even though the additional probes improve the coverage of regulatory elements, including 58 % of FANTOM5 enhancers, only 7 % distal and 27 % proximal ENCODE regulatory elements are represented. Detailed comparisons of regulatory elements from EPIC and WGBS show that a single EPIC probe is not always informative for those distal regulatory elements showing variable methylation across the region. However, overall data from the EPIC array at single loci are highly reproducible across technical and biological replicates and demonstrate high correlation with HM450 and WGBS data. We show that the HM450 and EPIC arrays distinguish differentially methylated probes, but the absolute agreement depends on the threshold set for each platform. Finally, we provide an annotated list of probes whose signal could be affected by cross-hybridisation or underlying genetic variation.

**Conclusion:**

The EPIC array is a significant improvement over the HM450 array, with increased genome coverage of regulatory regions and high reproducibility and reliability, providing a valuable tool for high-throughput human methylome analyses from diverse clinical samples.

**Electronic supplementary material:**

The online version of this article (doi:10.1186/s13059-016-1066-1) contains supplementary material, which is available to authorized users.

## Background

DNA methylation is the most well-characterised epigenetic mark in humans. It is defined as the addition of a methyl (CH_3_) group to DNA and in mammalian cells occurs primarily at the cytosine of cytosine-guanine dinucleotides (CpG). DNA methylation can modify the function of regulatory elements and gene expression and is therefore integral to normal human development and biological functioning. Perturbations to normal DNA methylation patterns can lead to dysregulation of cellular processes and are linked with disease. Widespread aberrations in DNA methylation are a well-established hallmark of many cancers [1] and a growing body of literature shows a role for DNA methylation in the aetiology of other complex human diseases including chronic kidney disease [2], type 2 diabetes [3] and neuropsychiatric disease [4].

A full understanding of the role of DNA methylation in health and disease requires the development of tools that can simultaneously measure DNA methylation across large portions of the genome. The current ‘gold standard’ technique for fine mapping of methylated cytosines is whole-genome bisulphite sequencing (WGBS) [5]. This is based on the treatment of genomic DNA with sodium bisulphite, which converts unmethylated cytosines to uracils while leaving methylated cytosines unchanged, followed by whole-genome sequencing [6]. WGBS has been successfully applied to a range of biological tissues and cell lines to provide a complete map of the ~28 million CpG sites in the human genome [7]. However, the high cost of this approach and significant technical expertise currently required to generate and process WGBS data means that it is not always the most feasible method to interrogate DNA methylation in large cohort studies.

In recent years, the Illumina Infinium BeadChips have provided a popular, user-friendly alternative. Like WGBS, this technology is based on sodium bisulphite conversion of DNA, but with subsequent single base resolution genotyping of targeted CpG sites using probes on a microarray. The advantage of the Infinium platforms is that they are easy to use, time-efficient and cost-effective and show good agreement with DNA methylation measurements from other platforms [8]. For a full comparison of the strengths and weaknesses of different DNA methylation profiling methods, including Infinium methylation arrays, MBDcap-Seq and reduced representation bisulphite sequencing (RRBS), see the recent review by Stirzaker and colleagues [5].

The Infinium methylation technology was first introduced with the HumanMethylation27K BeadChip (HM27) in 2008, which featured 25,578 probes predominantly targeting CpG sites within the proximal promoter region of 14,475 consensus coding sequence (CCDS) genes and well-described cancer genes [8]. Probes were preferentially designed to target CpG islands due to the established relationship between DNA methylation at promoter CpG islands and gene expression [8]. The 12-sample per array format and genome-wide span of HM27 represented a significant advance over previous methods, which were low-throughput and restricted to a small number of genomic loci. HM27 allowed researchers to explore the role of DNA methylation in carcinogenesis and identify cancer biomarkers [9] and for the first time perform large-scale ‘epigenome-wide association studies’ (EWAS), which revealed the associations between DNA methylation patterns and tobacco smoking [10], ageing [11] and other complex human phenotypes.

In 2011, the HM450 BeadChip superseded the HM27 BeadChip. The HM450 retained the 12-sample per array design and featured 485,577 probes, including probes targeting 94 % of the CpG sites on the HM27 [12]. The new content was selected after consultation with a consortium of DNA methylation researchers and comprised a more diverse set of genomic categories, including: CpG islands, shores and shelves, the 5′UTR, 3′UTR and bodies of RefSeq genes, FANTOM4 promoters, the MHC region and some enhancer regions [12]. The improved coverage, together with the high sample throughput, of the HM450 made it a popular tool for EWAS studies and for the generation of reference epigenomes, including the International Cancer Genome Consortium (ICGC) and the International Human Epigenome Consortium (IHEC). Notably, The Cancer Genome Atlas (TCGA) consortium used the HM450 platform to profile more than 7500 samples from over 200 different cancer types [5] and it is the platform of choice for large-scale epidemiological studies such as the ARIES study, which is analysing 1000 mother-child pairs at serial time points across their lifetime [13].

Although the HM450 has been widely embraced by the epigenetics research community, the technology initially presented some technical challenges. Foremost among these was the two probe types on the HM450. In order to assay the new genomic regions included on the HM450, probes with a different chemistry were added. However, the two probe types have a different dynamic range, reflecting potential bias in the DNA methylation measurements. Extensive discussion within the field led to the development of bioinformatics methods that now allow us to address the technical impact of the two probe designs, as comprehensively reviewed by Morris and Beck [14]. Additionally, both the HM27 and HM450 featured a proportion of probes that either hybridised to multiple regions of the genome or targeted genetically polymorphic CpGs [15–17]. However, the thorough identification and annotation of these probes means that we can now easily account for misleading measurements during processing. Finally, DNA methylation changes rarely occur in isolation and are more likely to affect contiguous genomic regions. It was therefore necessary to develop methods to accurately identify these differentially methylated regions (DMRs) from HM450 data. Today, a range of analytical packages is available to researchers for regional methylation analysis, for example [18–20]. In summary, methods for processing and analysis of Infinium methylation BeadChips have matured considerably over recent years and we as a community are now extremely proficient at handling this type of data.

The remaining concern with the HM450 platform was that the probe design missed important regulatory regions. Recent studies using other platforms such as WGBS have demonstrated that DNA methylation at regulatory enhancers can determine transcription and phenotypic variation, through modulation of transcription factor binding. Thus accurate quantification of DNA methylation at more regulatory regions is essential for our understanding of the role of DNA methylation in human development and disease. To meet this need, Illumina have recently released the Infinium MethylationEPIC (EPIC) BeadChip, with new content specifically designed to target enhancer regions [21]. The EPIC BeadChip contains over 850,000 probes, which cover more than 90 % of the sites on the HM450, plus more than 350,000 CpGs at regions identified as potential enhancers by FANTOM5 [22] and the ENCODE project [23]. The EPIC array promises to be an essential tool to further our understanding of DNA methylation mechanisms in human development and disease, in particular the DNA methylation landscape of distal regulatory elements. In this paper we perform a comprehensive evaluation of the new EPIC platform.

## Results

### General features of the Infinium platforms

The Infinium methylation platforms use bead technology for highly multiplexed measurement of DNA methylation at individual CpG loci on the human genome. Individual beads hold oligos comprising a 23 base address, to allow identification of their physical location on the BeadChip, and a 50 base probe. Probe sequences are designed to be complementary to specific 50 base regions of bisulphite converted genomic DNA with a CpG site at the 3′ end of the probe [8]. After hybridisation to bisulphite converted DNA, single-base extension of the probe incorporates a fluorescently labelled ddNTP at the 3′ CpG site to allow ‘genotyping’ of the C/T conversion that results from bisulphite conversion. The fluorescent signal is then measured. The proportion of DNA methylation at a particular CpG site (also called the methylation beta-value (β)) is then ascertained by taking the ratio of the methylated (C) to unmethylated (T) signal, using the formula: β = intensity of the methylated signal/(intensity of the unmethylated signal + intensity of the methylated signal + 100). A β-value of 0 represents a completely unmethylated CpG site and a β-value approaching 1 represents a fully methylated CpG site.

**Fig. 1:**
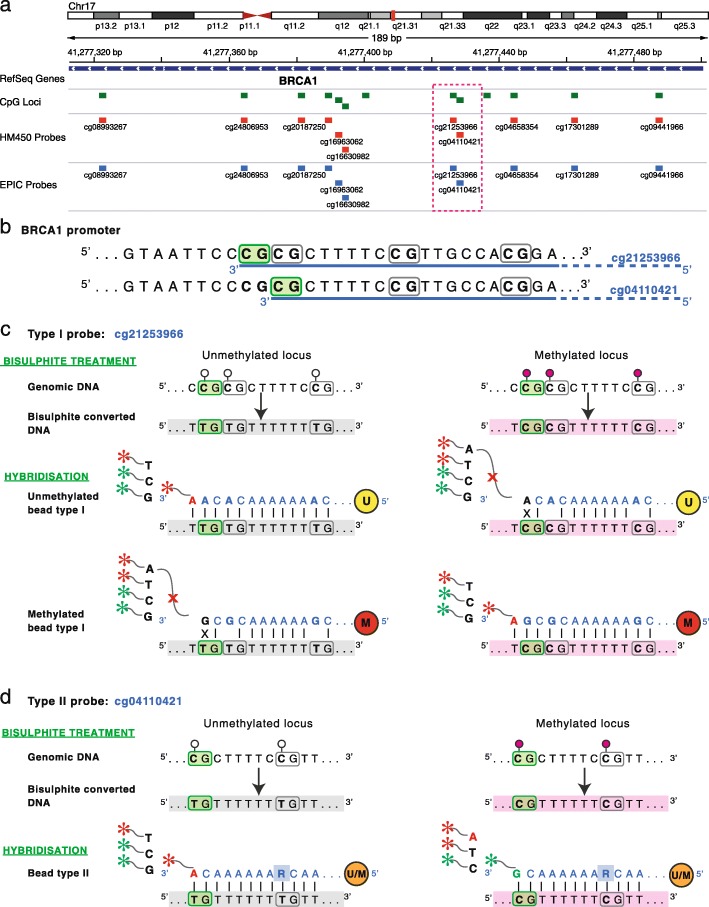
Infinium methylation probe design. **a** The difference in DNA methylation measurement process used by Illumina Infinium Type I and II probes is demonstrated with two probes targeting adjacent CpG sites in the *BRCA1* promoter. Both probes are present on EPIC and HM450 platforms. **b** Infinium I (cg21253966) and Infinium II (cg04110421) probes targeting two adjacent CpG sites in the *BRCA1* promoter region; the targeted CpG sites are highlighted in *green*. Each probe is designed to hybridise a 50 bp DNA sequence, underlined in blue, downstream of the targeted CpG site. **c** DNA methylation measurement with Infinium I probes is carried out by two beads – the unmethylated (U) bead measures the unmethylated signal and methylated (M) bead measures the methylated signal. The unmethylated signal detection for the cg21253966 probe is schematically represented on the *left panel*. Briefly, the unmethylated bead probe (U) sequence is designed to match bisulphite converted DNA sequence of the unmethylated locus. (Note that cytosines in both the target CpG site and all other CpG sites bound by the 50 bp probe are assumed to be unmethylated and therefore converted to Ts during bisulphite reaction.) The hybridisation of a bisulphite converted unmethylated DNA fragment to the bead enables single base extension and incorporation of a ddNTP labelled nucleotide matching the nucleotide immediately upstream of the target CpG site; in this case incorporation of an A nucleotide and signal detection in the *RED channel*. Hybridisation of the methylated bead probe (M), on the other hand, results in mismatch at the 3′ end of the probe and inhibition of single base extension. Detection of the methylated signal, shown on the *right panel*, follows similar steps. **d** For Infinium II probes the unmethylated and methylated signals are measured by the same bead (U/M). The bead probe sequence is designed to match bisulphite converted DNA of both the methylated and unmethylated locus. This is achieved by making the cytosine of the target CpG site the single base extension locus and replacing cytosines of all other CpG sites within the probe sequence with degenerate R bases that hybridises to both T (representing unmethylated and converted cytosine) and C (representing methylated and protected cytosine) bases. The unmethylated signal detection for the cg04110421 probe is schematically represented on the left panel. The hybridisation of the bisulphite converted unmethylated DNA fragment enables single base extension and incorporation of ddNTP labelled A nucleotide matching the unmethylated and converted cytosine at the target CpG site and signal detection on the *RED channel*. The detection of the methylation signal, shown on the *right panel*, is the same except that in this case single base extension results in incorporation of ddNTP labelled G nucleotide matching the methylated and protected cytosine at the target CpG site and signal detection on the *GREEN channel*

There are two types of probe design on the Infinium platforms. Type I probes have two separate probe sequences per CpG site (one each for methylated and unmethylated CpGs), whereas Type II probes have just one probe sequence per CpG site (Fig. 1). This means that, per CpG site assayed, Type II probes use half the physical space on the BeadChip compared with Type I. However, Type I probes are still necessary as their design characteristics mean they can measure methylation at more CpG dense regions than Type II probes. In this study we consider the distribution of probe types on the new EPIC array. However, their specific features and the technical issues resulting from having two different probe designs on the same platform have been discussed for the HM450 array in depth elsewhere and are beyond the scope of the current study [24–27].

### Design, genomic distribution and functional classification of probes on the EPIC array

**Fig. 2:**
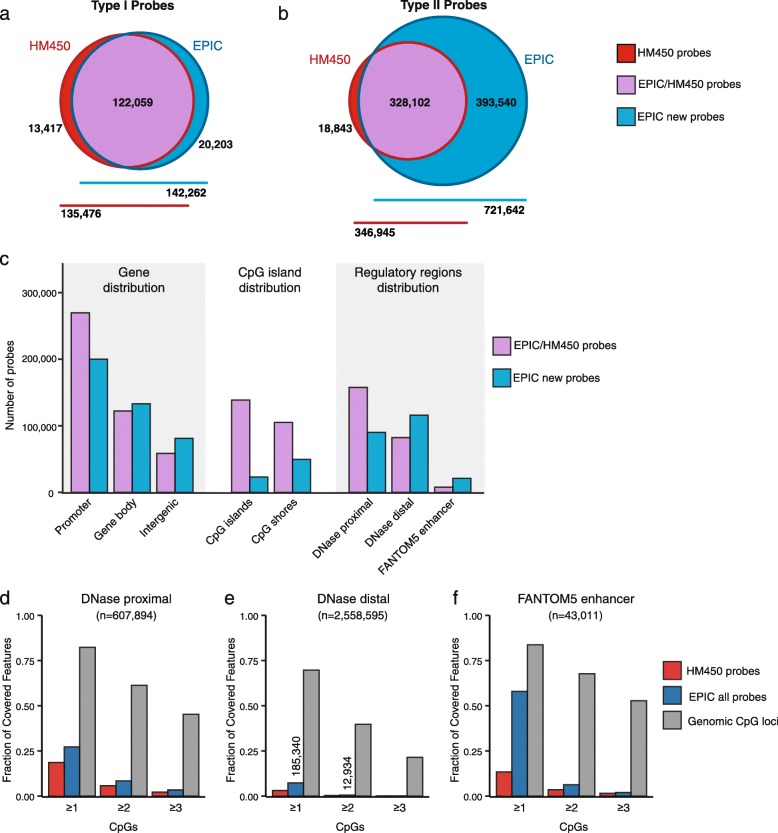
Distribution of probes on the HM450 and EPIC platforms. **a**, **b** *Venn diagrams* indicating overlap of (**a**) Type I and (**b**) Type II CpG probes on the HM450 and EPIC platforms. **c** Distribution of probes across different genome annotation categories: (1) GENCODE19 genes; (2) CpG islands; and (3) regulatory regions defined using ENCODE DNAse hypersensitivity sites and FANTOM5 enhancers. Probes are separated according to whether they are new to EPIC (‘EPIC new’, blue, *n* = 413,743) or common to HM450 and EPIC (‘EPIC/HM450’, purple, *n* = 450,161). **d**–**f** Fraction of (**d**) DNase proximal peaks, (**e**) DNase distal peaks and (**f**) FANTOM 5 enhancers which overlap more than one, two or three HM450 probes (*red*), EPIC probes (*blue*) or genomic CpG sites (*grey*)

To evaluate the new EPIC platform, we first compared the design, genomic distribution and functional classification of probes with those on the preceding HM450 BeadChip, using the manufacturer supplied annotation data (MethylationEPIC_v-1-0_B2 and HumanMethylation450_15017482_v-1-2 manifest files). The EPIC platform has probes targeting 866,836 cytosine positions on the human genome, of which 863,904 (99.7 %) are CpG dinucelotides and 2932 (0.3 %) CNG targets. Additionally, there are 59 probes targeting SNP sites to allow sample matching and 636 probes for sample-dependent and sample-independent quality control. Comparison with the HM450 annotation data shows that the EPIC includes 450,161 (93.3 %) of the HM450 CpG probes (Fig. 2a and b). Investigation of the 32,260 (6.7 %) HM450 CpG probes, excluded from the EPIC array showed that the excluded probes were enriched for Type I probes (odds ratio (OR) = 1.93, confidence interval (CI) = 1.89–1.98) and probes previously flagged as being unreliable (‘discard’) by Naeem et al. [17] (OR = 1.15, CI = 1.13–1.18), suggesting that Illumina excluded some of the least reliable probes on the HM450. We performed further analysis to identify the remaining HM450 and new EPIC probes whose signal could be unreliable due to cross-reactivity and underlying genetic variation. This revealed 43,254 cross-reactive probes with ≥ 47 bp homology with an off-target site, of which 15,782 (36.5 %) are new to the EPIC platform. We also identified overlap with genetic variant categories with minor allele frequency > 5 % at: (1) target CpG sites (*n* = 12,378); (2) single base extension sites of Type I probes (*n* = 772); and (3) overlapping the probe body (*n* = 97,345). For full-annotated lists, see Additional file 1: Table S1; Additional file 2: Table S2; Additional file 3: Table S3; Additional file 4: Table S4; Additional file 5: Table S5 and Additional file 6: Table S6.

The EPIC platform features 413,743 new CpG probes, of which 95 % (*n* = 393,540) are Type II probes (Fig. 2a and b). The high proportion of new Type II probes reflects the increased coverage of distal regulatory elements, which are largely CpG-sparse regions of the genome and so amenable to profiling by Type II probes. Type II probes also take up less physical space on the BeadChip, thus maximising probe number, however the number of samples measured per BeadChip was reduced from 12 on the HM450 to 8 on the EPIC.

To ascertain the genomic distribution of probes on the EPIC array, we next calculated the number of probes targeting promoters, gene body and intergenic regions using GENCODE V19 annotation data (Fig. 2c; Additional file 7: Table S7). EPIC probes are principally located at promoters (54 %), followed by gene bodies (30 %) and then intergenic regions (16 %). We then took a closer look at the distribution of new EPIC probes (new EPIC) as compared to probes that are common between EPIC and HM450 (EPIC/HM450). Interestingly, new EPIC probes show increased targeting of gene bodies—32 % of new EPIC probes (*n* = 133,021) versus 27 % of EPIC/HM450 probes (*n* = 122,158)—and intergenic regions—20 % of new EPIC probes (*n* = 80,902) versus 13 % of EPIC/HM450 probes (*n* = 58,507). Our next analysis revealed that 19 % and 18 % of all EPIC probes are located in CpG islands and CpG island shores, respectively. However, a much smaller fraction of new EPIC probes is allocated to these regions—6 % of new EPIC probes versus 31 % of EPIC/HM450 probes at CpG islands and 12 % of new EPIC probes versus 23 % EPIC/HM450 probes at CpG island shores. Both new EPIC and EPIC/HM450 probes are most commonly located in non-CpG island regions (341,069 (82 %) and 206,589 (46 %), respectively).

The large number of new EPIC probes targeting gene body, intergenic and non-CpG island regions is consistent with Illumina’s intention to include new content covering distal regulatory elements on the EPIC. To explicitly test this, we took advantage of several publicly available catalogs of regulatory elements, curated across a wide range of cell types [28–30]. Thurman et al. [28] used high-throughput profiling of DNase hypersensitive sites (DHSs) to identify regions of open chromatin that correspond to sites of transcription factor binding in place of canonical nucleosomes; the most recent update of this catalog [31] integrates DNase hypersensitivity assays across 177 cell types and contains 3,166,489 regulatory regions which are further subdivided into proximal (*n* = 607,894) and distal (*n* = 2,558,595) sites based on distance to GENCODE V19 transcription start sites [32]. We also included the FANTOM5 compendium of 43,011 transcribed enhancer regions identified through computational mining of CAGE-Seq transcription data from 432 primary cell, 135 tissue and 241 cell line human samples [29].

Using these publicly available catalogs we identified the EPIC probes targeting each type of regulatory region and observed an increase in the number of new EPIC probes targeting DNAse distal sites and FANTOM5 enhancers (Fig. 2c) (DNase distal new EPIC = 115,797 versus EPIC-HM450 = 82,168, FANTOM5 new EPIC = 21,070 versus EPIC-HM450 = 7763). Considering both the new EPIC and EPIC-HM450 probes together, we found that overall 27 % of DNAse proximal, 7 % of DNAse distal and 58 % of FANTOM5 enhancers were covered by probes on the EPIC array (Fig. 2d–f). Thus the proportion of all 607,894 DNAse proximal and 2,558,595 DNAse distal regions covered by the EPIC array was low. However, DNAse elements vary by cell type, so repeating the analysis for each cell type individually we found that the proportion of covered regulatory elements per cell type was in the range of 39–57 % (DNAse proximal) and 10–25 % of DNAse distal sites (for individual cell type statistics, see Additional file 8: Table S8). We then used the median number of occurrences of each DHS across the 177 cell types to subdivide the DHSs into those that are least frequently occurring (specific) and most frequently occurring (common) (Additional file 7: Figure S1a, b). Interestingly, we observe that probes on the EPIC array cover 17 % and 4 % of the specific DHSs and 38 % and 11 % of the common DHSs, for proximal and distal DHSs, respectively (Additional file 7: Figure S1c, d and Additional file 8: Table S8).

Of the regulatory regions covered, most are represented by just one probe on the array (Fig. 2d–f). For example, of the 185,340 DNAse distal sites targeted by probes on the EPIC array, 93 % (*n* = 172,406) are targeted by only one probe (see Fig. 2e). It is currently unknown if a single probe on the EPIC array can accurately capture methylation variation across the extent of a regulatory region, especially as regulatory regions are less CpG dense than CpG islands and can show abrupt methylation changes across the locus.

### Reproducibility of the EPIC array

To assess the performance of the EPIC array we ran a series of technical analyses using DNA from different samples types (cell lines, clinical samples and blood) commonly profiled in array-based methylation studies: a transformed prostate cancer cell line (LNCaP); primary cell cultures of prostate epithelial cells (PrEC); patient-matched cancer associated fibroblasts (CAF) and non-malignant tissue associated fibroblasts (NAF); and infant blood from archival Guthrie cards. We first profiled the DNA on both the HM450 and EPIC arrays. Initial quality control steps using the control SNP probes on the array confirmed correct sample matching and demonstrated the utility of these probes on the EPIC array (Additional file 7: Figure S2).

**Fig. 3:**
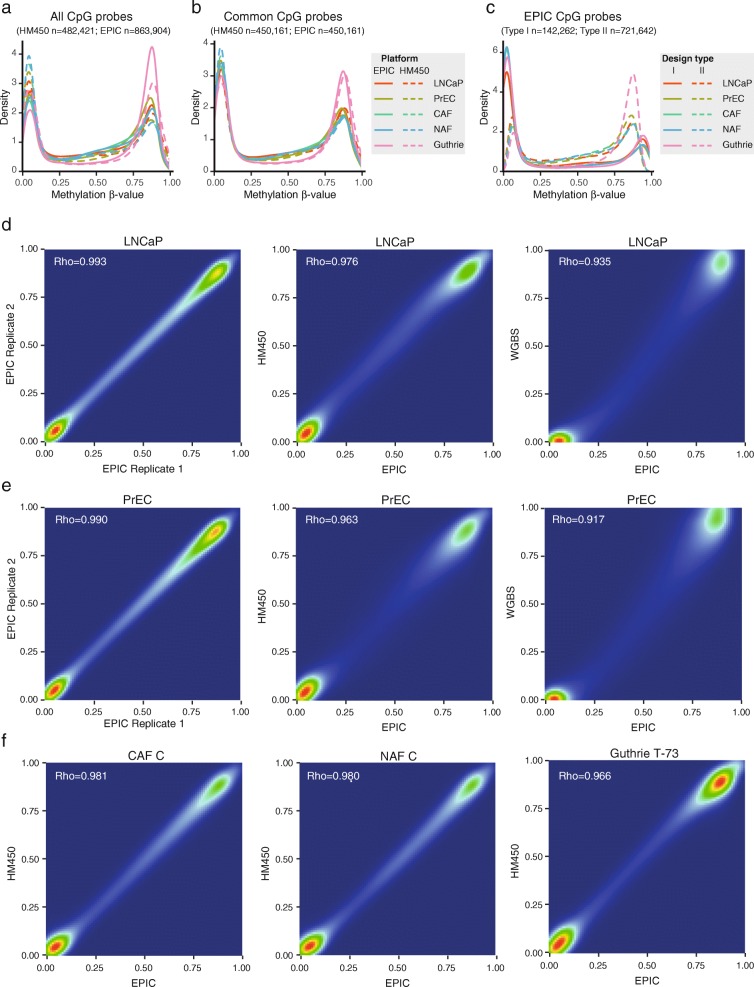
Comparison of methylation values on the HM450 and EPIC platforms. **a**, **b** *Density plots* of the methylation (beta) values for a subset of samples profiled on both the HM450 and EPIC platforms, showing (**a**) all CpG probes on the HM450 (*n* = 482,421) and EPIC (*n* = 863,904) and (**b**) only CpG probes that are common to HM450 and EPIC platforms (*n* = 450,161). **c** *Density plot* of methylation values for the same subset of samples on the EPIC platform, showing shift in methylation values between Type I and II probes. **d**–**f** *Scatter plots* show correlation between methylation measurements from different platforms: EPIC-EPIC, EPIC-HM450 and EPIC-WGBS for (**d**) LNCaP and (**e**) PrEC; and EPIC-HM450 for (**f**) CAF, NAF and Guthrie samples

DNA methylation β-value density plots showed that on both platforms all samples had a bimodal distribution, with the two peaks indicating unmethylated and fully methylated states typical of DNA methylation data (Fig. 3a). However, we noted that the unmethylated peak was higher than the methylated peak in the HM450 data, whereas the two peak heights were more similar in the EPIC data. This likely reflects the new probe content on the EPIC array, which (as described above) targets more intergenic, non-CpG island regions, which are often methylated. To confirm this, we recreated the density plots with only the probes common to both platforms (*n* = 450,161). As expected, this plot showed strong similarity between the methylation density distribution of HM450 and EPIC for each matched sample (Fig. 3b). Finally, we plotted the β-values from the EPIC array by Type I and Type II probes separately and found that the distribution of Type II probes was shifted relative to Type I, as frequently reported in the HM450 literature (Fig. 3c).

To determine the reproducibility of DNA methylation values of the same sample run on the EPIC array, we hybridised technical replicates of the LNCaP and PrEC cell lines on the same BeadChip. We found a high correlation between β-values of the two sets of technical replicates (Spearman rank correlation LNCaP *ρ* = 0.993; PrEC *ρ* = 0.990) (Fig. 3d and e). Next, to assess the performance of the EPIC array in comparison with other platforms we extended our comparison of matched samples run on the HM450 and EPIC array. Again Spearman rank correlation tests showed an extremely high correlation of β-values between the two platforms (LNCaP *ρ* = 0.976; PrEC *ρ* = 0.963; CAF C *ρ* = 0.981; NAF C *ρ* = 0.980; Guthrie card T-73 *ρ* = 0.966) (Fig. 3d–f; Additional file 7: Figure S3). These data indicate that the DNA methylation data generated from the EPIC array are extremely reproducible across platforms and, importantly, is amenable for integration with existing HM450 data. Finally, we compared EPIC DNA methylation values with matched whole genome bisulphite sequencing data (average coverage > X20), currently considered the gold-standard technique for measuring DNA methylation. Again we found a high correlation between platforms (LNCaP *ρ* = 0.935, PrEC *ρ* = 0.917) (Fig. 3d and e). This is especially notable as the WGBS and Infinium array DNA methylation values are derived from different types of raw data (continuous intensity values versus count-based reads, respectively, which makes the array measurements of DNA methylation less sensitive towards the extremes of 0 and 1).

### Reproducibility of differential analysis

**Fig. 4:**
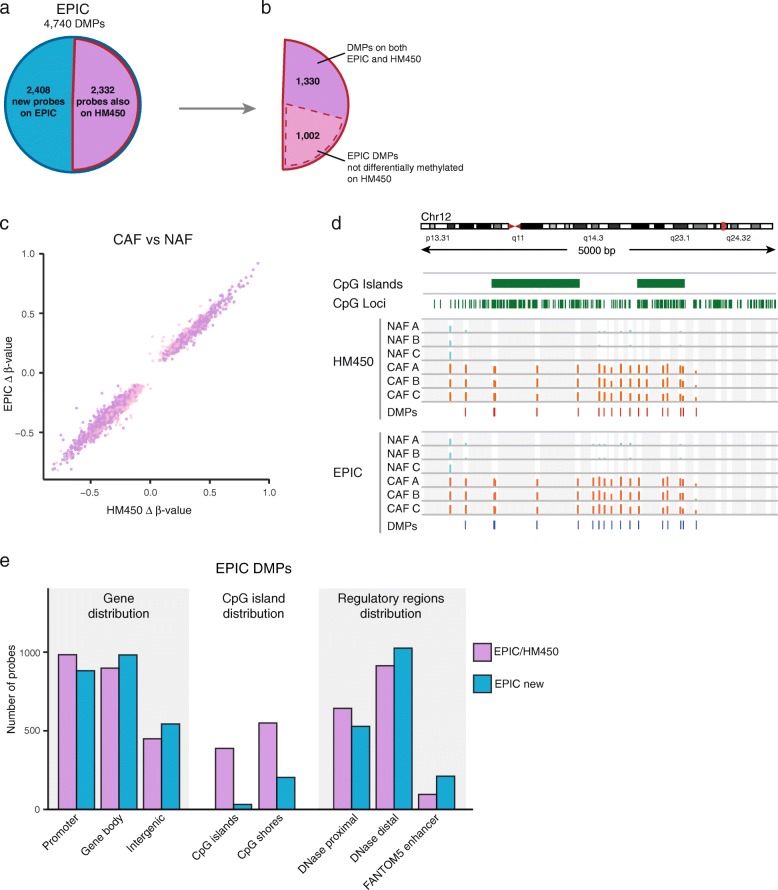
Reproducibility of CAF vs. NAF differential analysis across HM450, EPIC and WGBS platforms. **a** *Pie chart* indicating number of differentially methylated probes (DMPs) on the EPIC that are present on the HM450 array. **b** *Segmented pie chart* showing number of EPIC DMPs that are present on the HM450 and the proportion that are also called as DMPs using HM450 data. **c** *Scatter plot* showing strong agreement in the direction and magnitude of the estimated CAF-NAF methylation difference (Δ β**-**value) on the EPIC vs. HM450 at the EPIC DMPs. **d ** Genomic region densely covered by probes on the EPIC and HM450 arrays shows extensive differential methylation between CAF and NAF samples on both platforms. **e** Distribution of DMPs across different genome annotation categories: (1) GENCODE19 genes; (2) CpG islands; and (3) regulatory regions defined using ENCODE DNAse hypersensitivity sites and FANTOM5 enhancers. Probes are separated according to whether they are new to EPIC (*blue*, *n* = 2408) or common to HM450 and EPIC (*purple*, *n* = 2332)

Infinium methylation arrays are commonly used to identify loci that are differentially methylated between sample groups. To compare the ability of the HM450 and EPIC array to distinguish differentially methylated probes (DMPs), we used the limma package [33] to perform separate analyses on the two platforms and identified 4740 EPIC and 2054 HM450 differentially methylated probes (DMPs) between three matched pairs of CAFs and NAFs (unpaired analysis; *p* < 0.001; Δβ > 0.1; see ‘Methods’). Approximately half the EPIC DMPs are present as probes on the HM450 (2332/4740) (Fig. 4a). Of the 2332 common probes, ~57 % (*n* = 1330) are also called as differentially methylated on HM450 (see Fig. 4b). However, if we relax the *p* value cutoff for HM450 DMP calling to *p* < 0.01, the number of common probes that are DMPs on EPIC and HM450 is increased to ~94 % (2184). We also observed excellent overall agreement in estimated Δβ-values of EPIC and HM450 data (Spearman rank correlation *ρ* = 0.98, *p* < 2.2E-16) (Fig. 4c). An example of differential methylation called by both EPIC and HM450 platforms is shown in a genomic region spanning two CpG islands upstream of a gene promoter (Fig. 4d). The region is densely covered by probes and methylation data from both platforms reveal extensive hypermethylation in CAF samples. Interestingly, more than half of the EPIC DMPs are located in probes that are unique to the EPIC array (*n* = 2408) (see Fig. 4a) and a large fraction of these (*n* = 1026, 43 %) are located in distal regulatory elements (see Fig. 4e). This highlights the ability of the EPIC platform to interrogate and detect differential methylation in previously inaccessible loci, especially those located in regulatory regions.

### Ability of EPIC to detect differential methylation at distal regulatory elements

**Fig. 5:**
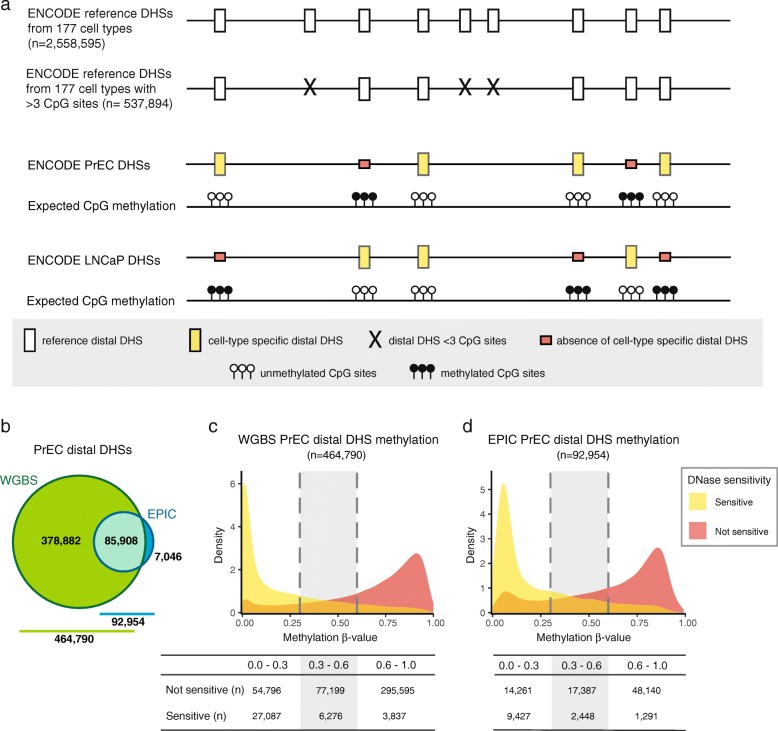
Overview of approach and assessment of DNA methylation at distal regulatory elements defined using ENCODE DNAse hypersensitivity data. **a** Outline of the approach taken to analyse the relationship between DNAse hypersensitivity and DNA methylation in LNCaP and PrEC cell lines, using a *schematic representation* of the genome. **b** *Venn diagram* showing the sites that were informative in PrEC WGBS and EPIC methylation data at ENCODE reference distal DHS sites. **c**, **d** Methylation status of DNAse sensitive and non-sensitive sites according to **c**) WGBS and **d**) EPIC PrEC methylation data

Several recent studies using whole-genome methylation profiling methodologies demonstrated the important role of DNA methylation in modulating transcription factor binding to regulatory elements of the genome at regions distal to transcription start sites [34, 35]. Therefore, the addition of regulatory regions on the EPIC array is an important advance. However, as detailed above, the majority of these regions are represented by only one probe on the array (Fig. 2d–f). To determine the ability of a single probe to capture the methylation status of an entire regulatory region, we compared EPIC to WGBS methylation data in LNCaP and PrEC cells across distal DHSs. Using an approach summarised in Fig. 5a, we considered all reference distal DHSs as defined across 177 cell lines by the ENCODE project [31]. To ensure that we had enough DNA methylation data for a meaningful analysis, we selected only the reference distal DHSs containing three or more CpG sites (*n* = 537,894). For each reference distal DHS, we then computed the mean methylation level of (1) all EPIC probes and (2) WGBS CpG loci to estimate the methylation status over the DHS region; for the WGBS data we only considered DHSs with 50X coverage. As shown in Fig. 5b, PrEC WGBS and EPIC data were informative for 464,790 (~86 %) and 92,954 (~17 %) reference DHSs, respectively, while LNCaP WGBS and EPIC data were informative for 495,591 (~92 %) and 92,912 (~17 %) reference DHSs, respectively (Additional file 7: Figure S4a).

As a first step to check the quality of the data, we tested whether DNA methylation at reference DHSs was associated with closed chromatin. More specifically, we used ENCODE DHS catalog annotation data to determine a subset of regions present in PrEC and LNCaP cell lines. Using this cell-type specific DHS data, we observed a strong negative relationship between the methylation status of reference distal DHSs and the presence of distal DHSs in both cell lines (Fig. 5c and d; Additional file 7: Figure S4b and c). Specifically, WGBS data show that the vast majority (~73 %; 27,087/37,200) of the assayed PrEC distal DHSs are lowly methylated (β ≤ 0.3) and only 3837 sites (~10 %) are extensively methylated (β > 0.6); log-odds ratio of 3.63 (95 % CI 3.60–3.67) (Fig. 5c). Similarly, most LNCaP distal DHSs assayed by WGBS are lowly methylated, 30,118 or ~67 % and just 6801 sites (~15 %) are extensively methylated; log-odds ratio of 2.49 (95 % CI 2.46–2.52) (Additional file 7: Figure S4b). The same relationship between methylation and DHS status is observed with the EPIC methylation data; PrEC log-odds ratio of 3.20 (95 % CI 3.14–3.26) and LNCaP log-odds ratio of 2.61 (95 % CI 2.56–2.66) (Fig. 5d; Additional file 7: Figure S4c).

**Fig. 6:**
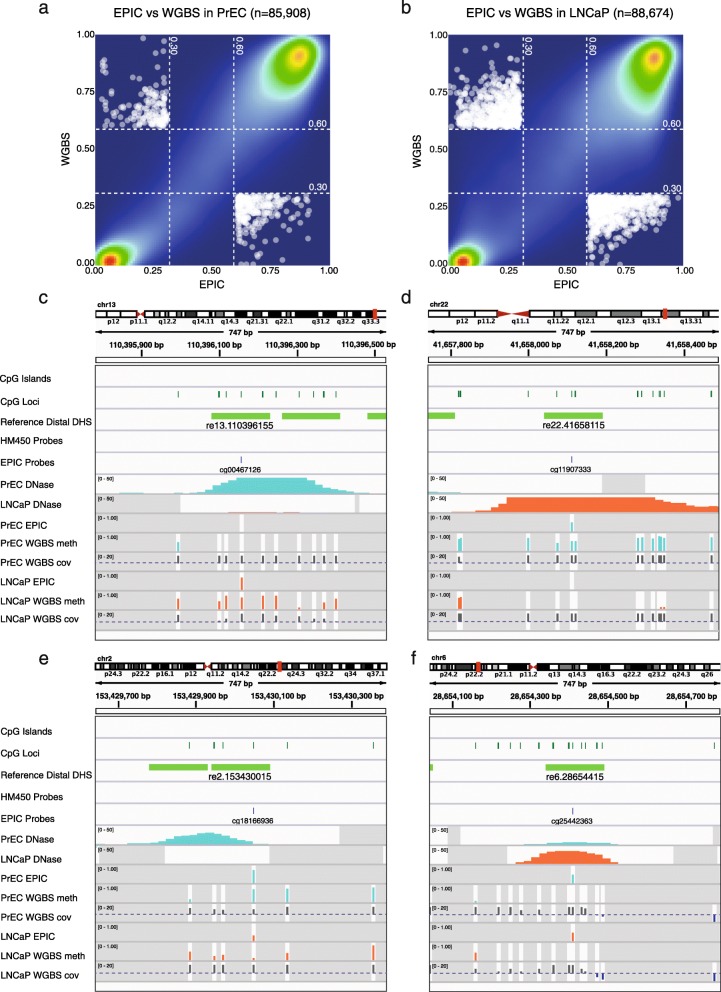
Ability of EPIC to detect differential methylation at distal regulatory elements defined using ENCODE DNAse hypersensitivity data. **a**, **b** *Scatter plot* showing overall agreement in DNA methylation between EPIC probes and WGBS across distal regulatory regions for (**a**) PrEC and (**b**) LNCaP. **c**–**f**. Comparison of DNA methylation between EPIC and WGBS across distal regulatory regions. *Tracks* show ENCODE DHS data across 177 reference cell lines and PrEC and LNCaP DHS data separately; EPIC and WGBS methylation measurements for PrEC and LNCaP; and WGBS coverage for each site, with the 10X threshold represented by a *dashed purple line* for reference. *Dark grey shading* indicates regions that were not assayed by each technology. **c** Genomic region shows agreement in DNA methylation between EPIC probe and WGBS across distal regulatory region re13.110396155. PrEC features a DNAse sensitive peak and low methylation, while LNCaP lacks DNAse sensitivity and has high methylation. **d** Genomic region shows agreement in DNA methylation between EPIC probe and WGBS across distal regulatory region re22.41658115. LNCaP features a DNAse sensitive peak and low methylation, while PrEC lacks DNAse sensitivity and has high methylation. **e** Genomic region shows disagreement in DNA methylation between EPIC probe and WGBS across distal regulatory region re2.153430015 due to probe positioning. PrEC features a DNAse sensitive peak and high methylation at the border of the peak where the EPIC probe is located, but low methylation in the centre of the peak (not covered by EPIC probes). **f** Genomic region shows disagreement in DNA methylation between EPIC probe and WGBS, in both LNCaP and PrEC samples, across distal regulatory region re6.28654415

Next, we performed a direct comparison of reference distal DHS methylation values from WGBS and EPIC PrEC data across DHSs common to both platforms (PrEC: 85,908, LNCaP: 88,674). Methylation readouts from the two platforms agree well with Spearman’s Rho correlation coefficients of 0.883 for PrEC and 0.822 for LNCaP (Fig. 6a and b). For PrEC and LNCaP, respectively, 87 % and 80 % of regions showed < 20 % difference between platforms; 61 % and 54 % showed < 10 % difference; and 33 % and 30 % showed < 5 % difference. For example, the reference DHS re13.110396155 (located ~10 kb upstream of the prostate cancer associated *IRS2* gene [36, 37]) presents as a DHS in PrEC but not in LNCaP, and accordingly, WGBS data show the region to be lowly methylated in PrEC and highly methylated in LNCaP. Crucially, we found that a single EPIC probe in the centre of the DHS accurately reflects the methylation status of the surrounding CpG sites (Fig. 6c). Figure 6d highlights another example of an agreement in DNA methylation readouts between the two platforms at a reference DHS re22.41658115 present in LNCaP but not PrEC cells. This DHS is located within the gene body of *RANGAP1*, which has previously been associated with signalling cascades in prostate cancer [38].

Notably, only a small number of DHSs (PrEC: 432 or ~0.5 %; LNCaP: 1377 or ~ 1.5 %) show large disagreements, i.e. lowly methylated (β ≤ 0.3) in WGBS and heavily methylated (β > 0.6) in EPIC or vice versa (Fig. 6a and b). Visual inspection of a subset of these ‘disagreement loci’, at reference DHSs present in a cell line and heavily methylated according to EPIC, revealed two common types of disagreement (Additional file 7: Figures S5 and S6). The first occurs when the methylation measurement of the EPIC probe is consistent with the WGBS methylation measurement at the single CpG site assayed, but due to probe positioning does not capture the variable methylation across the DHS (Fig. 6e). The second type of disagreement arises when the methylation measurement of the EPIC probe disagrees with the WGBS methylation at the single CpG site assayed, as well as the adjacent CpG sites, suggesting a technical artifact in the EPIC probe such as described for the HM450 array [15–17] (Fig. 6f).

## Discussion

We have performed a comprehensive analysis of the new EPIC methylation array and find it to be a robust and reliable platform. The EPIC array almost doubles the content of the preceding HM450 array, retaining the majority of HM450 probes, and provides valuable new content. Two types of probe chemistry are used on the Infinium HM450 and EPIC methylation arrays. The new probes on the EPIC are primarily Type II probes, which take up less physical space on the array and are suitable for targeting the less CpG dense regions of the genome. The increase in Type II probe measurements is associated with a shifted distribution of methylation values compared to the HM450. A number of methods to correct for this are already available [24–27] and we recommend that these should be utilised in data processing and interpretation of results. A subset of the probes on the array may have a confounded signal due to cross-reactivity or underlying genetic sequence variation. We have provided a full list of annotated probes to aid identification and filtering for EPIC array users in Additional file 1: Table S1; Additional file 2: Table S2; Additional file 3: Table S3; Additional file 4: Table S4; Additional file 5: Table S5 and Additional file 6: Table S6.

Comparison of matched samples run on EPIC and HM450 shows excellent agreement in methylation values and in the ability to detect sites of differential methylation between samples. The convincing cross-platform reproducibility paves the way for integration of new EPIC data with existing HM450 datasets. The reliability of the EPIC array for methylation evaluation is further shown through comparison between matched samples profiled on EPIC and WGBS. Even though the new content on the EPIC array is designed to target distal regulatory regions, the majority of regions are targeted by just one probe. Remarkably, we found that at the majority (~80 % of regions with a cross-platform difference < 20 %) of targeted distal regions the single EPIC probe accurately represents DNA methylation across the entire region. Where methylation at the EPIC probe did not represent the distal regulatory region, the probes were often located at CpG sites showing variable methylation compared to adjacent CpGs. An array platform will never be as comprehensive as WGBS, so researchers planning a more detailed investigation of regulatory regions would be advised to interrogate or validate methylation patterns across a critical region of interest using an independent technology.

## Conclusion

The EPIC array represents a significant improvement in genomic coverage compared to the HM450, in particular with a higher proportion of probes capturing methylation at enhancers; however, the proportion of distal regulatory elements interrogated is still limited and the methylation level of one CpG probe per element is not always reflective of the neighbouring sites. EPIC does, however, maintain many of the desirable features of the HM450, such as ease of analysis and affordability, which allows profiling of large sample numbers and integration with valuable data resources generated from existing HM450 datasets, to allow for new important insights in genomic regulation in disease states. As such, the new EPIC platform will ensure methylation arrays remain a central tool in epigenetic research while cost and complexity of bioinformatic analysis still prohibits the large-scale use of WGBS.

## Methods

### DNA samples

LNCaP prostate cancer cells were cultured as described previously [39]. Normal prostate epithelial cells were cultured according to the manufacturer’s instructions in prostate epithelial growth medium (PrEGM, catalogue no. CC-3166; Cambrex Bio Science) as described previously [40]. Genomic DNA for both cell lines was extracted using QIAamp DNA Mini and Blood Mini kit following the manufacturer’s protocol for cultured cells (Qiagen).

Three blood spot punches, each 3 mm in diameter, were taken from 5–7-year-old archived neonatal screening (Guthrie) cards from five children whose mothers participated in the DOMInO trial [41]. Written informed consent was obtained from the mothers to access their child’s newborn screening card for the purposes of isolating DNA for (epi)genetic studies. DNA was extracted using GenSolve technology (IntegenX) followed by purification using the QIAamp DNA micro kit (Qiagen) and an additional ethanol precipitation step. The quantity of the DNA samples was assessed using the Quant-iT Picogreen dsDNA assay (Life Technologies).

Patient-matched cancer associated fibroblasts (CAFs) and non-malignant tissue associated fibroblasts (NAFs) (*n* = 3 pairs) were isolated and validated as previously described [42]. DNA was extracted using the DNeasy kit (Qiagen) with on-column RNase A digestion. DNA quantity and quality was assessed using a NanoDrop 2000 and gel electrophoresis.

### Bisulphite conversion and Infinium arrays

DNA (250–750 ng) was treated with sodium bisulphite using the EZ DNA methylation kit (Zymo Research, CA, USA). For a full description of samples and replicates run on the arrays see Additional file 7: Figure S2. DNA methylation was quantified using the Illumina Infinium HumanMethylation450 (HM450) and HumanMethylationEPIC (EPIC) BeadChip (Illumina, CA, USA) run on an Illumina iScan System (Illumina, CA, USA) using the manufacturer’s standard protocol.

Raw IDAT files were processed with Illumina’s GenomeStudio software V2011.1 and background normalised using negative control probes to generate methylation β-values which were used for all downstream analyses. We used MethylationEPIC_v-1-0_B2 manifest for processing EPIC data and HumanMethylation450_15017482_v-1-2 for HM450 data. All downstream analysis was conducted using the hg19/GRCh37 human genome assembly.

### Whole genome bisulphite sequencing

WGBS libraries were prepared for LNCaP/PrEC using the Illumina Paired-end DNA Sample Prep Kit (Illumina, CA, USA). Briefly, DNA (1 μg) was spiked with 0.5 % unmethylated lambda DNA (Promega) in a final volume of 50–65 μL. DNA was sheared to 150–300 bp by sonication with a Covaris S2. Library preparation was performed according to the manufacturer’s protocol; fragments were end-repaired and adenylated before ligation of Illumina TruSeq adaptors. Gel size selection (260–330 bp) was used to purify and size select the ligated DNA, using Qiagen Gel extraction kit (Qiagen, part #28704) and DNA was eluted in 20 μL H2O. Bisulphite treatment was carried out as previously described [43] with the bisuphite reaction performed for 4 h at 55 °C. After bisulphite cleanup, the DNA pellet was resuspended in 50 μL H_2_O. The adaptor-ligated bisulphite-treated DNA was enriched by performing five independent polymerase chain reactions (PCRs) for ten cycles using PfuTurboCx Hotstart DNA polymerase (Stratagene) in a volume of 50 μL per PCR. The five independent PCRs were pooled together, cleaned up using the MinElute PCR purification kit and eluted in 20 μL Qiagen EB buffer. Library quality was assessed with the Agilent 2100 Bioanalyzer using the High-sensitivity DNA kit (Agilent, CA, USA). DNA was quantified using the KAPA Library Quantification kit by quantitative PCR (KAPA Biosystems). Paired-end 100 bp sequencing was performed for each library on the Illumina HiSeq 2500 platform using Truseq v3 cluster kits and SBS kits.

Bisulphite reads were aligned to the human genome using version 1.2 of an internally developed pipeline, publicly available for download from http://github.com/astatham/Bisulfite_tools. Briefly, adaptor sequences and poor quality bases were removed using Trimgalore (version 0.2.8, http://www.bioinformatics.babraham.ac.uk/projects/trim_galore/) in paired-end mode with default parameters. Bismark v0.8.326 was then used to align reads to hg19 using the parameters ‘-p 4 –bowtie2 –X 1000 –unmapped –ambiguous –gzip –bam’. PCR duplicates were removed using Picard v1.91 (http://broadinstitute.github.io/picard). Count tables of the number of methylated and unmethylated bases sequenced at each CpG site in the genome were constructed using bismark_methylation_extractor with the parameters ‘-p –no_overlap –ignore_r2 4 –comprehensive –merge_non_CpG –bedgraph –counts –report –gzip –buffer_size 20G’. The PrEC and LNCaP libraries had a total of 908,201,217 and 1,271,535,305 reads, respectively. Both libraries passed basic quality control checks with 88 %/87 % alignment rate, ×20/×26 mean coverage and 99.7 %/99.7 % bisulphite conversion for PrEC/LNCaP.

### Public data

ENCODE DNAse hypersensitivity data were downloaded from ENCODE data portal http://www.encodeproject.org/data/annotations/v2 [31] in June 2015. We obtained a master list of distal DNase peaks comprising 2,558,595 regions and list of proximal DNase peaks comprising 607,894 regions. We also obtained DNase signal data for PrEC (ENCODE accession ENCFF001EEC) and LNCaP (ENCODE accession ENCFF001DWI) cell lines.

FANTOM5 compendium of enhancer elements was downloaded from FANTOM5 enhancer data portal http://enhancer.binf.ku.dk/presets/[29] in November 2015. We obtained a list of permissive enhancers comprising 43,011 regions.

CpG island coordinates were obtained from UCSC browser. CpG island shores were obtained from CpG island coordinates by taking 2 kb flanking regions and subsequently removing any overlaps with CpG islands.

GENCODE v19 transcript annotations were downloaded from GENCODE data portal ftp://ftp.sanger.ac.uk/pub/gencode/Gencode_human/release_19 [32]. Promoter regions were defined as regions of +/–2 kb around transcription start sites (TSSs). Gene body regions were defined as transcripts plus 2 kb flanking upstream and downstream regions, minus the promoter regions defined above. Intergenic regions were defined as regions of the genome not overlapping gene body or promoter regions.

Phase 3 variant data from the 1000 Genomes project were downloaded in August 2016: ftp://ftp.1000genomes.ebi.ac.uk/vol1/ftp/release/20130502/ALL.wgs.phase3_shapeit2_mvncall_integrated_v5b.20130502.sites.vcf.gz [44].

### Data analysis

All analyses were conducted in the R statistical software (Version > = 3.2.2).

#### Coverage computations

For each annotation region we computed the number of overlapping HM450 probes, EPIC probes and CpG loci. The regions were then stratified based on the number of overlaps: one or more overlaps (≥1), two or more overlaps (≥2) and three or more overlaps (≥3).

#### Identification of probes overlapping genetic variants

The Bioconductor ‘VariantAnnotation’ package was used to parse the 1000 Genomes VCF file and extract all ‘SNP’ and ‘INDEL’ variants overlapping EPIC probes. We examined variant position with respect to EPIC probe coordinates to further separate the variants into three categories: (1) variants overlapping targeted CpG sites; (2) variants overlapping single base extension sites for Infinium Type I probes; and (3) variants overlapping the rest of the EPIC probe, 48 base pairs for Infinium Type I probes and 49 base pairs for Infinium Type II probes. Results were filtered to only include genetic variants with a maximum minor allele frequency >0.05.

#### Identification of cross-reactive probes

We followed the written description in [16] to identify regions of potential cross-reactivity using the BLAT alignment tool [45]. For EPIC probes that were common to the HM450, we were able to reproduce Chen et al.’s results with 99.998 % precision and 99.883 % recall (True positive: 1,281,444; False positive: 23; False negative: 1497—BLAT matches from probe sequences common to both platforms). We then extended this protocol to include all new probes on EPIC. Probe sequences with equal homology to more than one in silico genome are reported as ties. BLAT results are reported as zero-based coordinates.

#### Comparison to WGBS data

To compare EPIC DNA methylation readouts at single CpG loci to WGBS, we only considered EPIC targeted CpG sites with × 10 or more coverage in WGBS data. Given the high average coverage of our PrEC (LNCaP) WGBS data, more than 95 % (96 %) of EPIC probes were included in the comparison.

#### Differential methylation

We used the limma Bioconductor package [33] to perform differential methylation analysis between CAF and NAF samples on HM450 and EPIC datasets. We only considered probes for which there is a reliable methylation readout (detection *p* value < 0.01) in all six samples. We then transformed β-values into M-values using logit transformation: $M=lo{g}_2\left(\frac{\beta }{1-\beta}\right)$. (To avoid extreme M-values the β-values were capped at 0.01 and 0.99.) Standard limma workflow with unpaired contrast was then applied to computed M-values to call HM450 and EPIC differentially methylated probes.

#### Methylation status of distal DHS elements

For this analysis, we only considered distal DHS elements overlapping 3 or more CpGs (*n* = 537,894). For WGBS data, we computed average methylation levels for distal DHS regions with X50 or more coverage to reduce readout variability due to insufficient coverage. The average methylation level of a region was computed as the ratio of the number of unconverted CpGs (C readouts) to the total number of CpGs (C and T readouts) from all the WGBS reads overlapping the region. For EPIC data, we computed average methylation levels for distal DHS regions by averaging β-values for all probes overlapping the region; we used a single technical replicate from each sample. Only probes with robust signal intensities (detection *p* value < 0.01) were used. Thus for our analysis we had valid methylation values for 495,591 (or 92 %) regions from LNCaP WGBS data, for 464,790 (or 85 %) regions from PrEC WGBS data, for 92,912 (or 17 %) regions from LNCaP EPIC data and 92,954 (or 17 %) regions from PrEC EPIC data. We used ENCODE DHS annotation data to identify subset of DHSs with 3 or more CpGs present in PrEC and LNCaP cell lines. There are 40,469 sites present in PrEC with 37,200 sites interrogated by WGBS and 13,166 sites by EPIC. There are 47,616 sites present in LNCaP with 44,930 sites interrogated by WGBS and 13,921 sites by EPIC.

## Additional files

Additional file 1: Table S1. Cross-reactive probes on the EPIC array. Tabulated counts per probe of potentially cross-reactive regions (with ≥ 47 base pair homology). (CSV 97 kb)
Additional file 2: Table S2. Genomic regions complementary to cross-reactive probes on the EPIC array with ≥ 48 base pair homology. Individual BLAT hits corresponding to the cross-reactive regions in Additional file 1: Table S1. Zero-based coordinates are used. Hits homologous with the reverse complement have been oriented in the forward direction, to be consistent with the annotation in the Illumina manifest file. Reads map to either forward methylated (FM), forward unmethylated (FU), reverse methylated (RM), reverse unmethylated (RU), forward methylated and unmethylated (FMU), or reverse methylated and unmethylated (RMU) in silico versions of the genome. (CSV 28778 kb)
Additional file 3: Table S3. Genomic regions complementary to cross-reactive probes on the EPIC array with 47 base pair homology. Individual BLAT hits corresponding to the cross-reactive regions in Additional file 1: Table S1. Zero-based coordinates are used. Hits homologous with the reverse complement have been oriented in the forward direction, to be consistent with the annotation in the Illumina manifest file. Reads map to either forward methylated (FM), forward unmethylated (FU), reverse methylated (RM), reverse unmethylated (RU), forward methylated and unmethylated (FMU), or reverse methylated and unmethylated (RMU) in silico versions of the genome. (CSV 46885 kb)
Additional file 4: Table S4. Probes overlapping genetic variants at targeted CpG sites. (CSV 1336 kb)
Additional file 5: Table S5. Probes overlapping genetic variants at single base extension sites for Infinium Type I probes. (CSV 45 kb)
Additional file 6: Table S6. Probes with genetic variants overlapping the body of the probe: 48 base pairs for Infinium Type I probes and 49 base pairs for Infinium Type II probes. (CSV 11599 kb)
Additional file 7: Table S7. Supplemental Table and Figures. (PDF 1627 kb)
Additional file 8: Table S8. Distribution of EPIC probes across the regulatory regions of individual cell types defined using ENCODE DNAse hypersensitivity data. (XLS 46 kb)

## Abbreviations

CAF: Cancer associated fibroblast
CCDS: Consensus coding sequence
CpG: Cytosine-guanine dinucleotide
DHS: DNAse hypersensitivity site
DMP: Differentially methylated probe
DMR: Differentially methylated region
DRE: Distal regulatory element
EPIC: MethylationEPIC BeadChip
EWAS: Epigenome-wide association study
HM27: HumanMethylation27K BeadChip
HM450: HumanMethylation450 BeadChip
ICGC: International Cancer Genome Consortium
IHEC: International Human Epigenome Consortium
LNCaP: Lymph node carcinoma of the prostate
NAF: Non-malignant tissue associated fibroblast
PrEC: Prostate epithelial cell
RRBS: Reduced representation bisulfite sequencing
TCGA: The Cancer Genome Atlas
TSS: Transcription start site
WGBS: Whole-genome bisulfite sequencing
