# Supplementary material for: Critical evaluation of the Illumina MethylationEPIC BeadChip microarray for whole-genome DNA methylation profiling
Source: Genome Biol. 2016 Oct 7;17:208. doi: 10.1186/s13059-016-1066-1 (PMC5055731; doi:10.1186/s13059-016-1066-1)

Additional file 7: Table S7 and Figures S1-6

Table S7 Distribution of probes that are new to EPIC (EPIC new) or common to EPIC and HM450 (EPIC/HM450) across different genome annotation categories: i) GENCODE19 genes, ii) CpG islands and iii) regulatory regions defined using ENCODE DNAse hypersensitivity sites and FANTOM5 enhancers.

Figure S1 a-b) Distribution of the number of appearances of a) proximal and b) distal DHS across the 177 cell types. Dashed vertical line indicates the median number of appearances of a DHS across the 177 cell types. DHSs with less than the median number of occurrences are termed “specific” and those greater than the median are termed “common”. c-d) Proportion of specific and common c) proximal and d) distal DHSs covered by EPIC array probes

Figure S2 Control single nucleotide polymorphism (SNP) probes on the HM450 and EPIC array correctly group samples by the individual that they were derived from. Heatmap represents β-values for each sample and each SNP (rs) probe showing that samples group by the genotype of the individual, independent of whether they were measured on the EPIC or HM450 platform.

Figure S3 Comparison of methylation values on the HM450 and EPIC platforms for a-d) Guthrie, and e-h) pair-matched CAF and NAF samples.

Figure S4 Overview of approach and assessment of DNA methylation at distal regulatory elements defined using ENCODE DNAse hypersensitivity data.

a. Venn diagram showing the sites that were informative in LNCaP WGBS and EPIC methylation data at ENCODE reference distal DHS sites.

b-c. Methylation status of DNAse sensitive and non-sensitive sites according to c) WGBS and d) EPIC LNCaP methylation data.

Figure S5 Genomic regions show PrEC DNAse hypersensitivity sites (DHSs) with low methylation measured by WGBS (β ≤ 0.3), but high methylation according to the EPIC array (β > 0.6). The 6 sites showing the largest such disagreement between EPIC and WGBS measurement are shown. Disagreement in methylation measurements between platforms is either due to positioning of the EPIC probe within the DHS (a&f), or incorrect measurement of a single CpG site by the EPIC due to a technical artifact (b-e). Tracks show ENCODE DHS data across 177 reference cell lines, and PrEC and LNCaP DHS data separately; EPIC and WGBS methylation measurements for PrEC and LNCaP; and WGBS coverage for each site. Dark grey shading indicates regions that were not assayed by each technology.

Figure S6 Genomic regions show LNCaP DNAse hypersensitivity sites (DHSs) with low methylation measured by WGBS (β ≤ 0.3), but high methylation according to the EPIC array (β > 0.6). The 6 sites showing the largest such disagreement between EPIC and WGBS measurement are shown. Disagreement in methylation measurements between platforms is either due to positioning of the EPIC probe within the DHS (b-d&f), or incorrect measurement of a single CpG site by the EPIC due to a technical artifact (a&e). Tracks show ENCODE DHS data across 177 reference cell lines, and PrEC and LNCaP DHS data separately; EPIC and WGBS methylation measurements for PrEC and LNCaP; and WGBS coverage for each site. Dark grey shading indicates regions that were not assayed by each technology.

Supplementary Table 7

	Annotation	EPIC/HM450 (n=450,161)	EPIC/HM450 (%)	EPIC new (n=413,743)	EPIC new (%)	Total EPIC (n=863,904)	Total EPIC (%)
**i. GENCODE19**	Promoter	269,466	60	199,804	48	469,270	54
	Gene body	122,158	27	133,021	32	255,179	30
	Intergenic	58,507	13	80,902	20	139,409	16
**ii. CpG islands**	CpG islands	138,535	31	23,063	6	161,598	19
	CpG island shores	105,037	23	49,611	12	154,648	18
**iii. ENCODE regulatory regions**	DNase proximal	157,640	35	90,048	22	247,688	29
	DNase distal	82,168	18	115,797	28	197,965	23
	FANTOM5 enhancers	7,763	2	21,070	5	28,833	3

Supplementary Figure 1

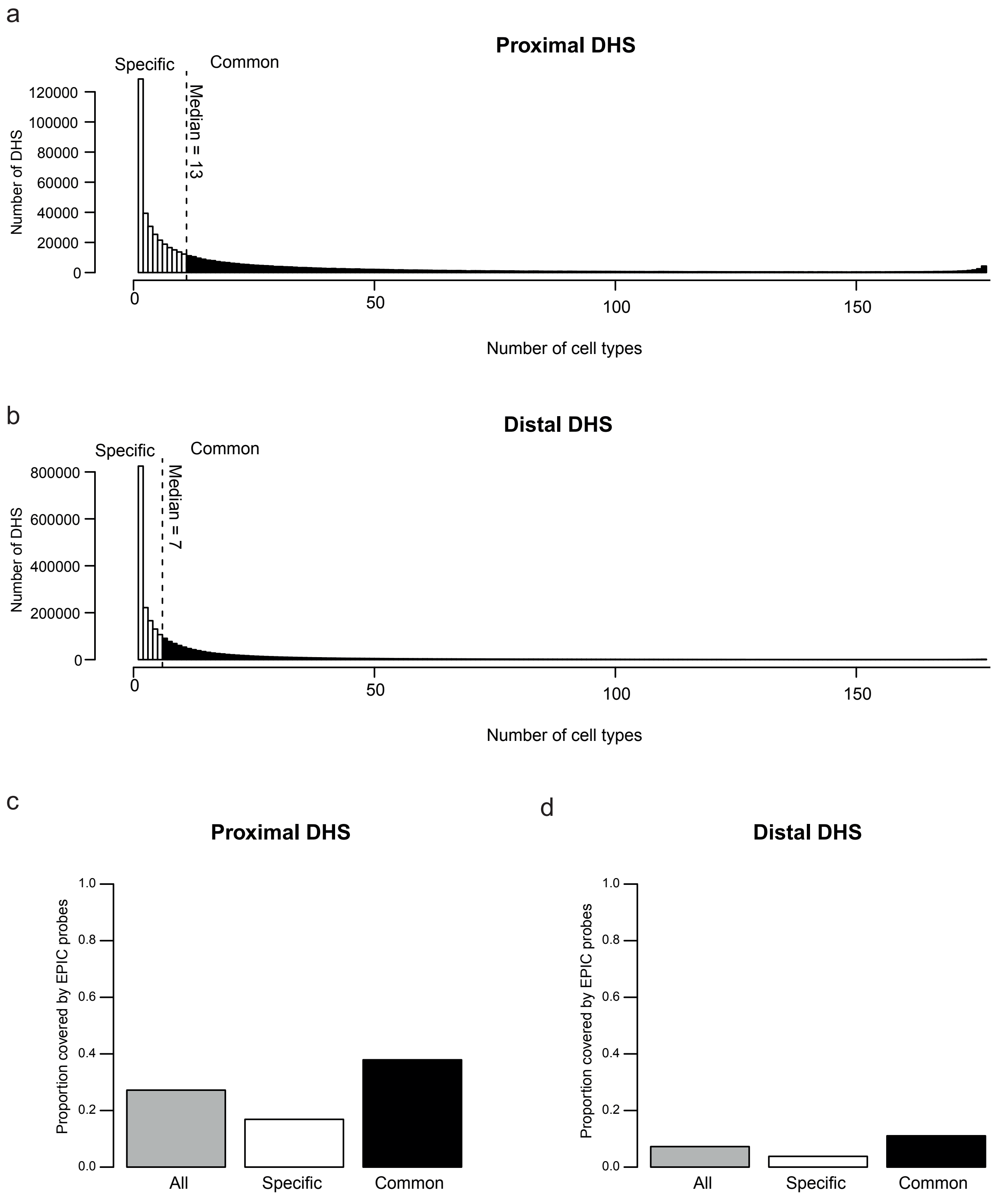

Supplementary Figure 2

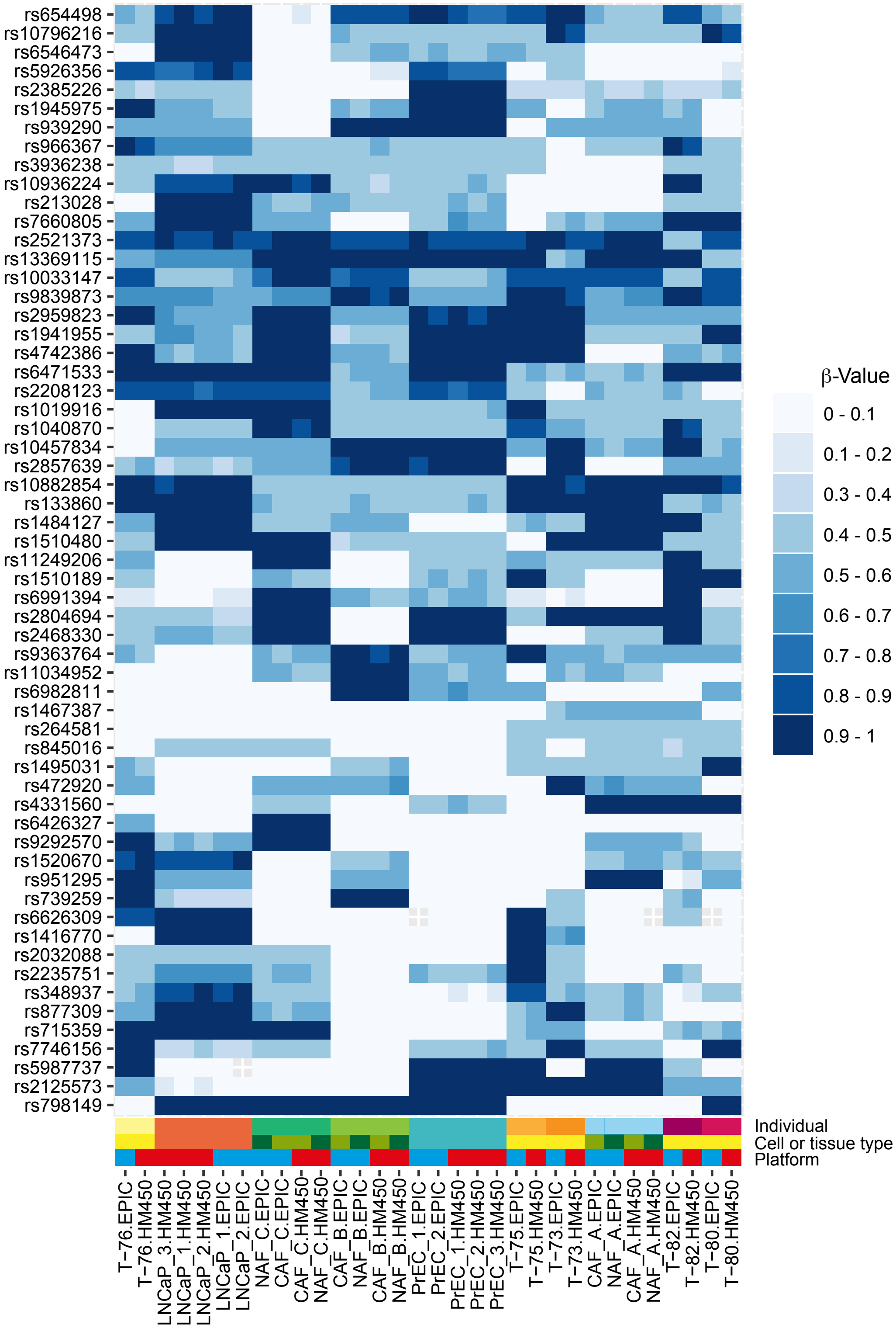

Supplementary Figure 3

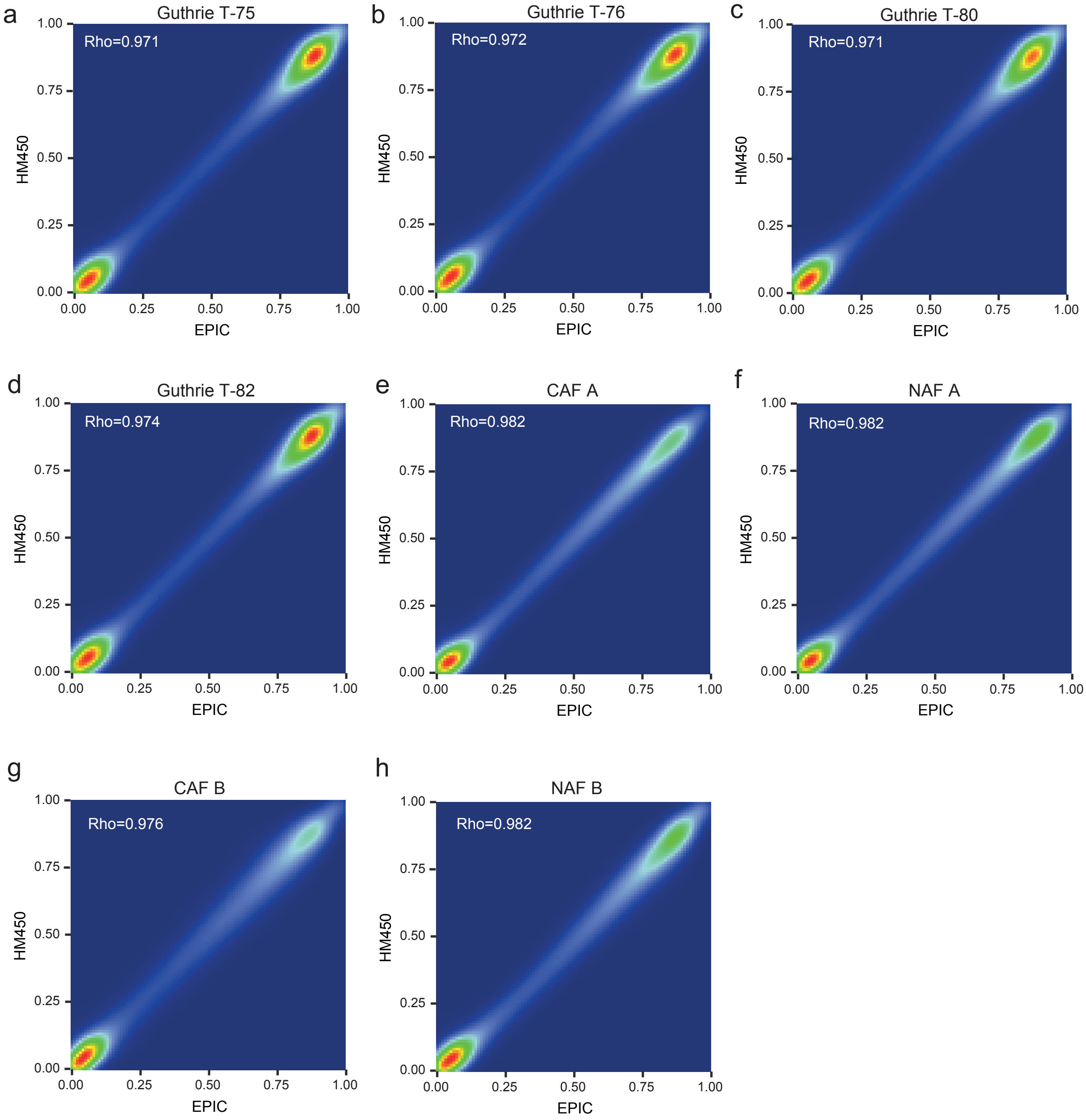

Supplementary Figure 4

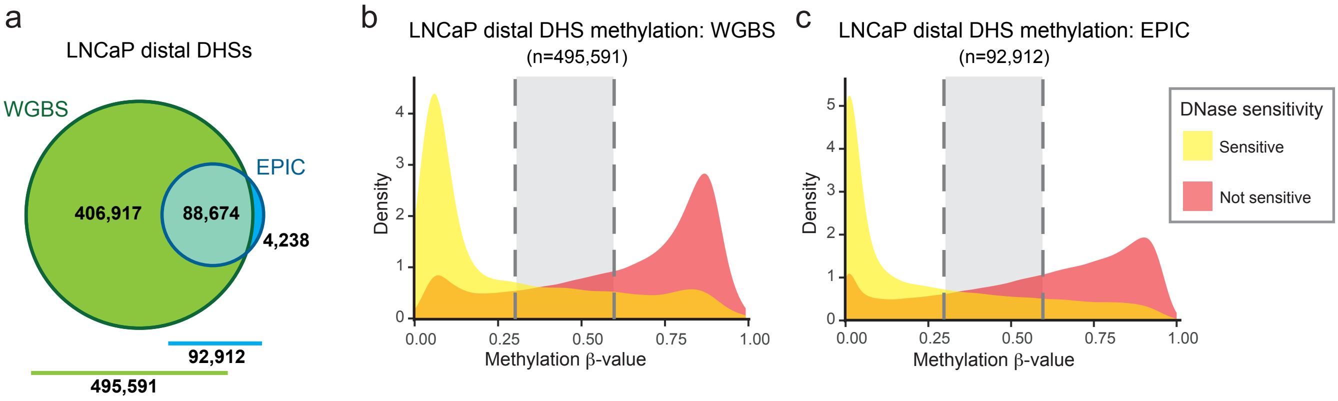

	0.0 - 0.3	0.3 - 0.6	0.6 - 1.0
Not sensitive	90,759	112,166	247,736
Sensitive	30,118	8,011	6,801

0.0 - 0.3	0.3 - 0.6	0.6 - 1.0
13,759	16,965	48,267
9,123	2,462	2,336

Supplementary Figure 5

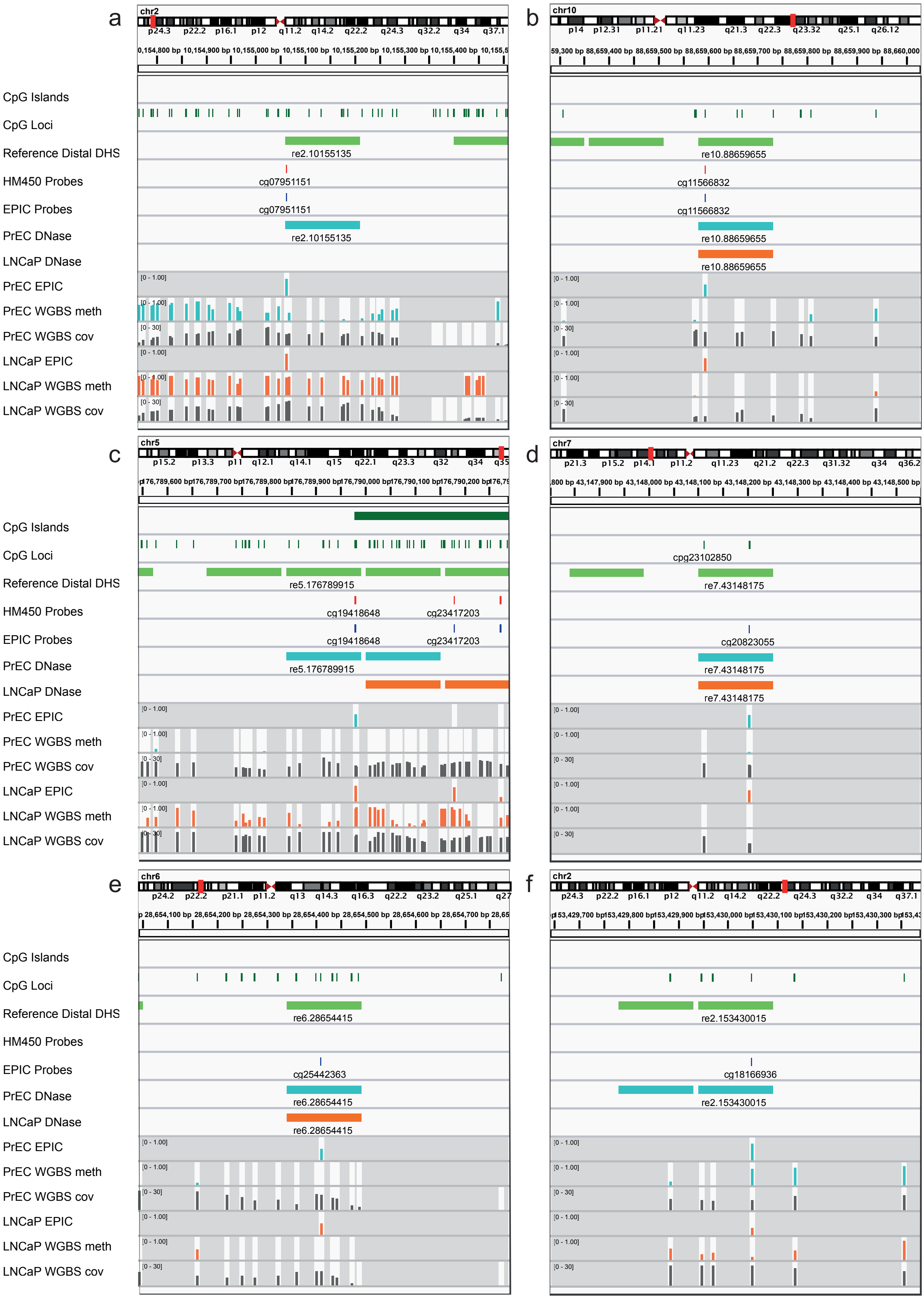

Supplementary Figure 6

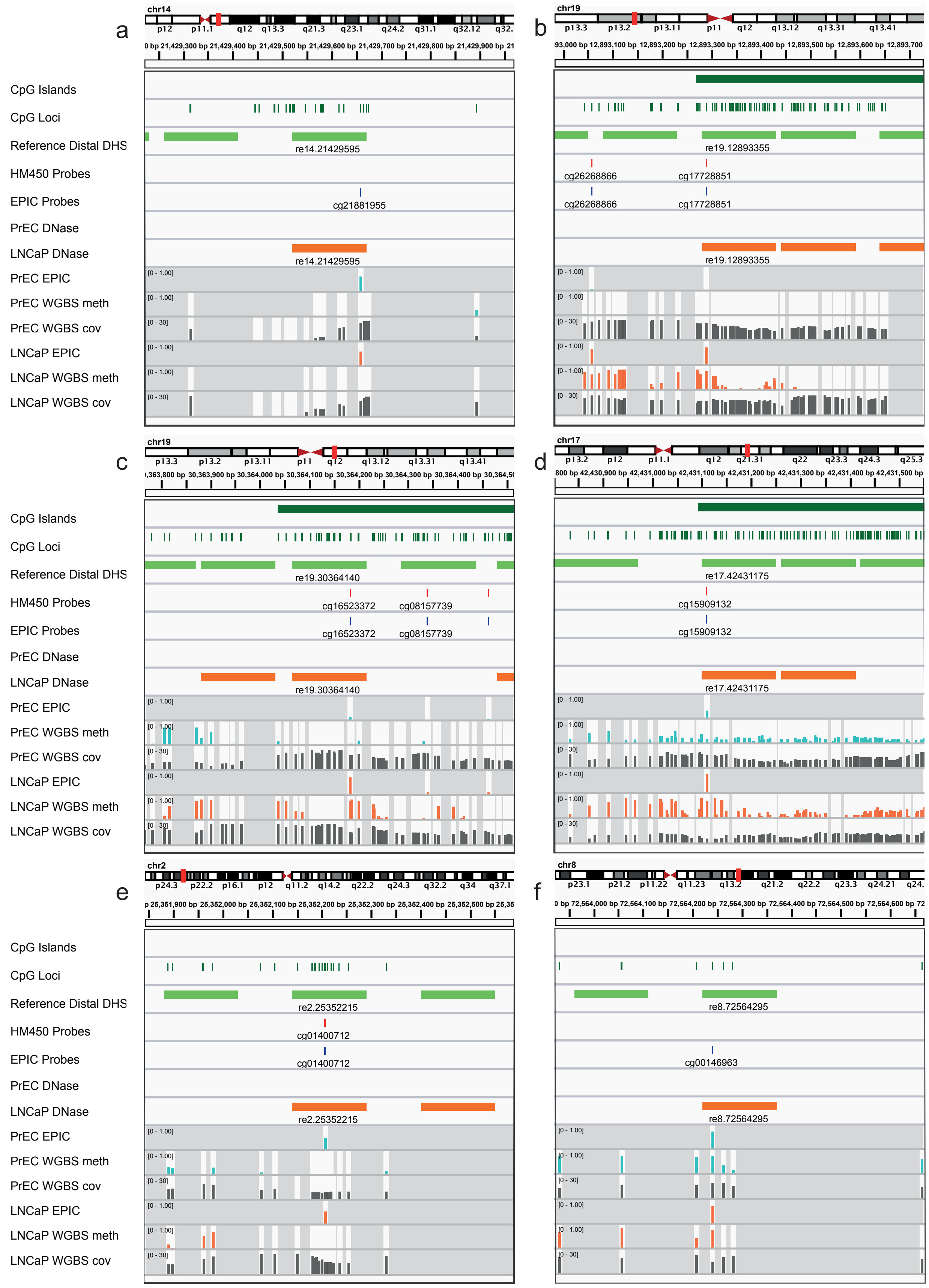

Supplement: Additional file 7: Table S7. — Supplemental Table and Figures. (PDF 1627 kb) [file 13059_2016_1066_MOESM7_ESM.pdf]
